# Funding for Postbariatric Body-Contouring (Bariplastic) Surgery in England: A Postcode Lottery

**DOI:** 10.1155/2014/153194

**Published:** 2014-03-20

**Authors:** Samrat Mukherjee, Sachin Kamat, Samuel Adegbola, Sanjay Agrawal

**Affiliations:** ^1^Bariatric Surgery Unit, Homerton University Hospital, Homerton Row, Homerton, London E9 6SR, UK; ^2^Centre for Digestive Diseases, Blizard Institute, Queen Mary University of London, London E1 2AT, UK

## Abstract

*Background.* With the increase in bariatric surgery in the UK, there has been a substantial increase in patients undergoing massive weight loss (MWL) seeking postbariatric body-contouring (bariplastic) surgery. However, there is a wide variation of availability on the National Health Service (NHS). *Aims.* To (1) review the funding policies of Primary Care Trusts (PCTs) in England for bariplastic surgery and (2) analyse the number of procedures funded in two consecutive financial years. *Methods.* We sent out questionnaires to all PCTs in England regarding their funding policies for bariplastic surgery and requested the number of procedures funded in 2008-09 and 2009-10. *Findings.* 121/147 (82%) PCTs replied to our questionnaires. 73 (60%) excluded all bariplastic procedures. 106/121 (87.6%) PCTs had referral guidelines for plastic surgery. 46/121 (38%) PCTs provided the total number of funded abdominoplasty-apronectomy (A-A) in the two financial years: total number of A-A applicants rose from 393 to 531, but approval for funding fell from 24.2% to 19.6%. Only 3 (2%) PCTs indicated increase in their future spending on bariplastic procedures in the next 5 years, with 67% planning to decrease or unsure about future funding. *Conclusion.* There exists a postcode lottery for bariplastic surgery in England and we feel the need for guidelines on provision of bariplastic procedures following MWL.

## 1. Introduction

Paralleling the increasing prevalence of obesity, there has been an exponential rise in bariatric surgery worldwide. A recent report showed a 70% rise in bariatric procedures in England in 2009-10 compared to 2008-09 [1]. Following bariatric surgery, most patients lose about 60–70% of their excess body weight in the initial 2 years. However, their skin does not contract with this massive weight loss (MWL) [2]. It often leads to disheartening ptotic skin envelopes which cause intertriginous infections, struggles with hygiene, impairment of mobility, interference with sexual intimacy, and personal distress because of appearance. These often lead to psychosocial problems which negatively affect the patients' quality of life [3] and may also hamper further weight loss or even lead to weight regain [4]. The term “bariplastic surgery” was proposed by Joseph O'Connell to encompass all body contouring procedures after bariatric surgery like apronectomy, abdominoplasty, mastopexy, brachioplasty, and buttock and thigh lift [5]. It resides at a perplexing intersection between cosmetic and functional surgery. Bariplastic surgery addresses the problems of redundant skin and has also shown to improve the quality of life and body image in these unique subgroup of patients [6–8].

In England, the National Health Service (NHS) is a publicly funded healthcare system and treatment on the NHS is free of cost at the point of delivery, but spending is presently controlled by the Primary Care Trusts (PCTs). Each PCT serves patients within a defined geographical area and decides independently how to allocate its budget. Bariplastic surgery is often regarded by the PCT as cosmetic and therefore of low priority, which means funding is either unavailable or subject to various criteria as determined by the PCT. In this age of economic constraints and rationing within the modern NHS, it is widely acknowledged that such treatments would be less available to patients on the NHS. As the PCTs make these difficult decisions independently of each other, treatments that are available in one trust may not be available in another.

The “Action on Plastic Surgery” (AoPS) document was produced by the NHS Modernisation Agency in 2005 (in association with the British Association of Plastic, Reconstructive and Aesthetic Surgeons, BAPRAS) [9]. This document provides guidance for commissioners of plastic surgery services regarding explicit criteria for referral and treatment thresholds for plastic surgical procedures that should be available on the NHS (Index 1).

*Index 1*. (AoPS guidance on who should be offered abdominoplasty-apronectomy (A-A)).

*AoPS recommends that A-A may be offered to the following groups of people*:

- those who are undergoing treatment for morbid obesity and have excessive skin folds,
- previously obese patients who have achieved significant weight loss and have maintained their weight loss for at least two years,
- stable BMI between 18 and 27 Kg/m^2^,

and

(iv) suffering from severe functional problems with the following:
  - recurrent intertrigo between the skin folds,
  - activities of daily living, for example, ambulatory problems,
  - surgical scarring leading to poor appearance and resulting in disabling psychological distress,
  - poorly fitting stoma bags.

The aims of this study were (1) to review the funding policies of PCTs in England for bariplastic surgery; (2) to evaluate the degree to which the AoPS guidelines are followed; and (3) to look at the total number of bariplastic procedures funded in England in the two consecutive financial years, 2008-09 and 2009-10. We also asked PCTs about their plans for expenditure on bariplastic surgery in the next 5 years. This study is the first comprehensive review of funding policies for bariplastic surgery in England.

## 2. Methods

We sent out a detailed questionnaire electronically to all 147 PCTs in England asking about their funding criteria for bariplastic surgery and whether they used the AoPS guidelines while drafting their policies. We also asked for the number of bariplastic procedures being funded in the two consecutive financial years, 2008-09 and 2009-10. We requested information on abdominoplasty, breast reduction and augmentation, facelift, and buttock, arm, and thigh lift postbariatric surgery (see the Supplementary Material available online at http://dx.doi.org/10.1155/2014/153194).

Many PCTs were part of regional groups that had been established to develop and manage policies such as funding criteria. Where a reply clearly represented a group policy, its data was applied to all PCTs in that group. In the cases where a PCT replied with its own policy and also was part of a group, its own policy was recorded.

PCTs that did not respond by 8 weeks were followed up with reminder emails and also by telephone wherever possible. The freedom of information act (2000) (FOI) was used where the information was withheld. The data was collected over ten months, from February to November 2011. Data was collated and analysed on Microsoft Excel spreadsheet.

## 3. Results

Out of 147 PCTs, responses were received from 121 (82.3%) PCTs. 4 PCTs acknowledged our request but refused to use the FOI to provide us with the data.

**Figure 1:**
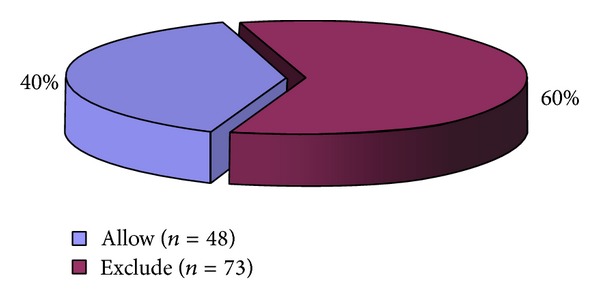
Pie chart showing the proportions of Primary Care Trusts (*n* = 121) that allow or exclude bariplastic surgery on the National Health Service.

Of the 121 PCTs, 48 (40%) PCTs fund bariplastic surgery on the NHS. The remaining 73 (60%) PCTs excluded all bariplastic surgery (Figure 1). Of these, 67 PCTs included a disclaimer for “exceptional circumstances” (EC); that is, individual patients could apply for “exceptional” funding and be considered for these procedures even if their guidelines excluded them from that particular treatment. The remaining 6 PCTs do not fund bariplastic procedures under any circumstances.

**Figure 2:**
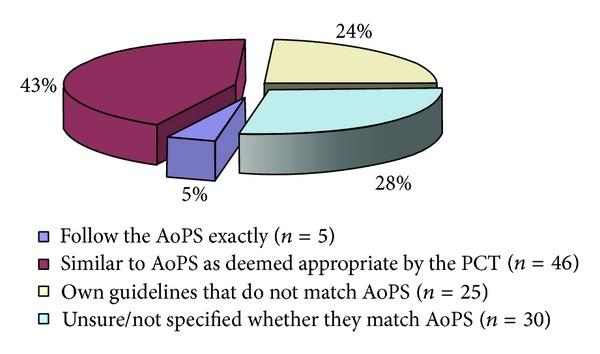
Pie chart showing the proportion of Primary Care Trusts (*n* = 106) having guidelines for plastic surgery procedures. AoPS is “Action on Plastic Surgery” document.

106 out of 121 (87.6%) PCTs had a guideline for plastic surgery procedures. Of these, 5 PCTs (4.7%) followed the AoPS guidelines exactly and 46 PCTs (43.4%) had guidelines similar to it. Of the rest, 25 PCTs (23.6%) had their own guidelines that did not match the AoPS and 30 PCTs (28.3%) were unsure or did not specify in the reply whether they followed the AoPS (Figure 2).

**Figure 3:**
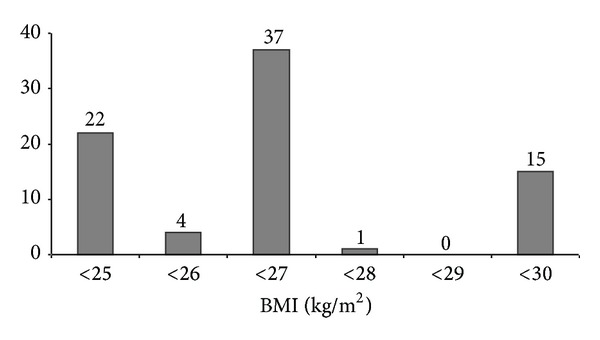
Chart showing specific body mass index (BMI) targets set by Primary Care Trusts (PCTs) (*n* = 79) that must be reached by patients seeking abdominoplasty-apronectomy.* y*-axis is the number of PCTs. The AoPS recommends a BMI < 27 Kg/m^2^.

**Figure 4:**
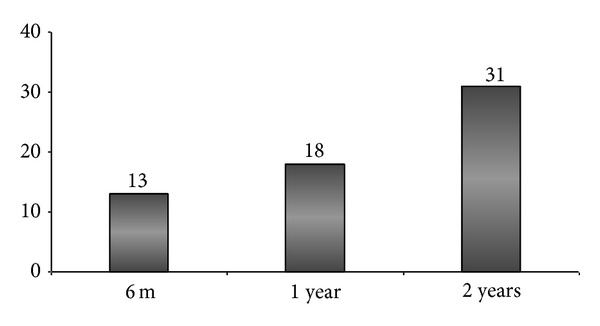
Chart showing length of time the Primary Care Trusts (PCTs) (*n* = 62) required patients to maintain their weight loss before being eligible for abdominoplasty-apronectomy.* y-*axis is the number of PCTs.

The AoPS guidelines mention 27 Kg/m^2^ as the upper limit of BMI for abdominoplasty-apronectomy (A-A). Of the 106 PCTs which had guidelines for A-A, 79/106 (74.5%) had specific body mass index (BMI) targets, ranging from 25 Kg/m^2^ to 30 Kg/m^2^, which the patient needed to achieve to be eligible for the procedure (Figure 3). Only 8/106 (7.6%) PCTs acknowledged the fact that, in the postbariatric surgery patients, it is difficult to attain the target BMI, because of the weight of the apron itself, and have put in a criterion of 50% excess weight loss to be eligible for A-A. The AoPS guidelines mention that the patients should have maintained their weight loss for at least 2 years following MWL. 62/106 (58.5%) PCTs required the weight loss to be maintained for a duration ranging from 6 months to 2 years before undergoing bariplastic surgery. This is summarised in Figure 4. The remaining 44 PCTs did not specify any timescale criteria.

**Figure 5:**
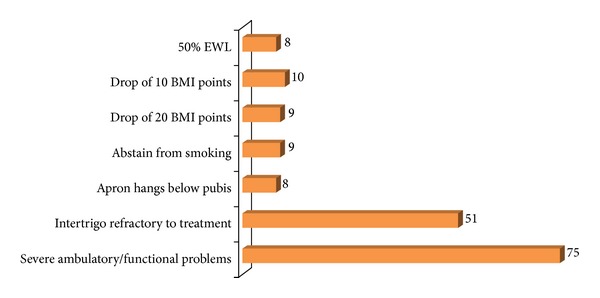
Chart showing the number of Primary Care Trusts that use a variety of additional assessment criteria to determine eligibility for patients requesting abdominoplasty-apronectomy. EWL = excess weight loss; BMI = body mass index.

75/106 (70.7%) PCTs specifically required the patients to have functional problems in the form of ambulatory difficulties, and 51/106 (48.1%) PCTs required the patients to have troublesome intertrigo, refractory to medical treatment from a period ranging from 6 weeks to 1 year. 8/106 (7.5%) PCTs required the abdominal apron to hang below the symphysis pubis and 9 (8.5%) required the patients to stop smoking to be allowed the A-A. Specific conditions imposed by the PCTs are shown in Figure 5.

**Figure 6:**
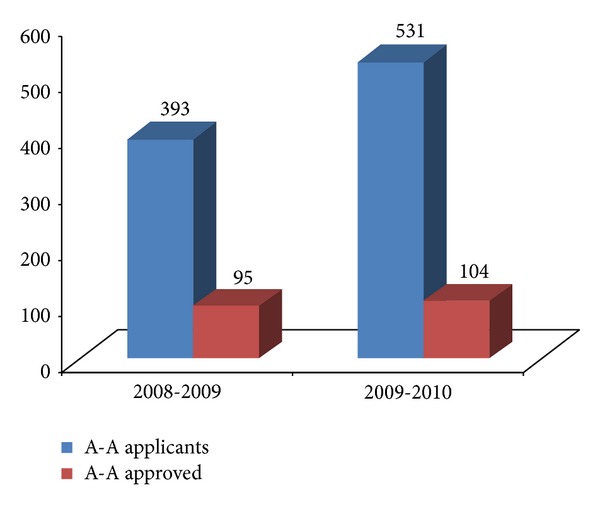
Chart showing current trend of abdominoplasty-apronectomy (A-A) being funded across England.* y*-axis is the number of patients.

The most common bariplastic procedure performed was A-A. The PCTs did not provide analyzable data on the other bariplastic procedures like mastopexy, brachioplasty, and buttock and thigh lift. 46/121 (38%) PCTs provided the total number of funded A-A in the two financial years (2008-09 and 2009-10). The total number of A-A applicants rose from 393 to 531, but the approval rate for funding fell from 24.2% to 19.6% consecutively in the two financial years (Figure 6).

**Figure 7:**
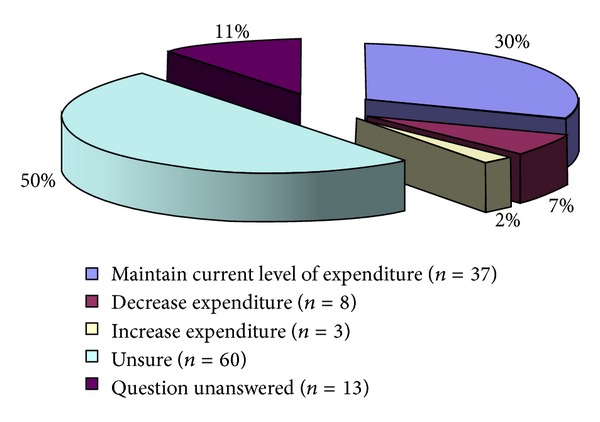
Pie chart showing the expenditure plans of the Primary Care Trusts for bariplastic surgery in the next five years.

On future service provision and expenditure, only 3/121 (2%) PCTs indicated that they would increase their spending on bariplastic procedures in the next 5 years, with 37/121 (31%) maintaining the current level of spending and the rest (67%) either planning to decrease or unsure about the future level of funding (Figure 7).

## 4. Discussion

There is very little in the literature on the prevalence of bariplastic surgery after bariatric surgery. According to a study by Mitchell et al. 33, out of 70 (47%) patients underwent bariplastic procedures after gastric bypass surgery [10]. Gusenoff et al. reported 11.3% of their 926 patients undergoing a body contouring procedure after gastric bypass [11]. A similar study from Austria suggested 21% of 252 patients had undergone body contouring surgery at least 1 year after gastric bypass surgery [12]. Assuming a conservative estimate of 20% needing bariplastic surgery, there would be a need for commissioning of more than 1600 body-contouring procedures in 2012-13 based on 8000 bariatric surgeries performed in the year 2010-11 in the UK [13]. This means large numbers of MWL patients will present for bariplastic surgery in the coming years.

Despite successful weight loss following bariatric surgery, the patients' body image and psychological state may actually deteriorate following the soft tissue deflation noted with MWL [3, 14]. Bariplastic surgery has been shown to improve the quality of life and body image of patients who have undergone MWL and optimize results achieved from bariatric surgery [6–8, 15]. As with burn surgery or cancer reconstruction, the body contouring procedures should be viewed as aesthetic as well as functional procedures. However, its role is still underestimated and often viewed as a cosmetic adjunct to bariatric surgery by the funding bodies [11].

The PCTs act independently of each other while drawing up their guidelines for the purposes of rationing. This leads to variability in funding for procedures in different regions within the NHS. Wraight et al., in 2007, showed a variation in guidelines across Trusts in the UK, amounting to a “postcode lottery” for patients seeking breast reduction [16]. More recent work by Henderson in 2009 [17] and Goodson et al. in 2011 [18] has shown that there does exist a disparity between PCTs for plastic surgery procedures, despite national guidelines. This has also been demonstrated for in vitro fertilisation (IVF) treatment in a report in 2009 [19]. In our study, 73/121 (60%) PCTs excluded all bariplastic procedures. It is also evident from our survey that majority (101/106, 95.3%) of PCTs have their own guidelines and individual cut-off points for referrals leading to a postcode lottery for bariplastic surgery. This is specially highlighted by the target BMIs and timescales following surgery set by the individual PCTs for patients seeking A-A.

At the present time there is no clear BMI cut-off above which bariplastic surgery should not be performed, but higher BMIs have been associated with increased complications [20]. A-A is generally avoided in those with a BMI > 35 Kg/m^2^ for these reasons [2, 21, 22]. The AoPS sets the upper limit of BMI as 27 Kg/m^2^ for A-A, but stricter criteria are being set by many PCTs as a means of rationing. Of the 79 PCTs who had specific BMI targets, 26 (32.9%) PCTs had criteria stricter than AoPS, but 15 (18.9%) PCTs have set a higher BMI cut-off at 30 Kg/m^2^ and acknowledge the fact that it might be difficult for these patients to lose further weight because of the intrinsic weight of the abdominal apron itself. In fact, Dafydd et al. have shown that the weight of the abdominal pannus in patients undergoing MWL may affect their BMI and exclude them from bariplastic procedures. They have therefore recommended that these patients should be considered for surgery even if their BMI is within 1-2 BMI points from the cut-off [23].

The ideal timing for the bariplastic procedure is when the weight loss has plateaued, the catabolism has ceased, and there has been an improvement in the comorbidities [24]. However, if a patients' weight loss has plateaued, waiting beyond 18 months before bariplastic procedures can sometimes be counterproductive [21]. The AoPS recommends that A-A could be offered to previously obese patients who have achieved significant weight loss and maintained their weight loss for at least two years. In our study we noted that, of the 62 PCTs who had specific criteria relating to timing of the A-A, 18 (29%) PCTs allow A-A in patients in whom the weight loss has been maintained for 1 year and 13 (21%) PCTs have reduced the timing to 6 months only leading to a wide variation in England.

Bariplastic surgery should be regarded as an integral component of the total care of the obese patient [2]. The National Institute for Health and Clinical Excellence (NICE) guidelines recommend that patients undergoing bariatric surgery should have information on, or access to, plastic surgery (such as A-A) where appropriate [25]. A recent survey of bariatric surgeons in the UK revealed that 41% reported that their patients did not have access to a plastic surgeon and a further 37% reported restricted access dependent on locally determined criteria. 96% felt that bariplastic surgery should be funded on the NHS in selected cases [14].

In our study 46 PCTs provided us with the actual number of A-A funded in consecutive years. The number of A-A funded was 95 and 104 in the two consecutive years which means at most on average each PCT was funding about 2 A-A in a year. This means a huge proportion of MWL patients are being denied A-A even at this present moment.

A recent survey from the UK, commissioned by the BAPRAS, shows that, of 1000 GPs questioned, 45% support NHS funding for bariplastic surgery [26]. We propose that postbariatric surgery patients should have easier access to the bariplastic procedures more in keeping with the pathway followed by breast cancer patients who are automatically funded for their oncoplastic procedures. It would be interesting to see if the changes in the NHS with the abolition of PCTs and the introduction of GP led clinical commissioning groups have any effect on the existing “postcode lottery” for these procedures in England in the future.

Our study had a response rate of 82% which is a very good response for this type of questionnaire study and provides a good estimation of the current postcode lottery for these procedures in England. Our questionnaire looked into the funding criteria for bariplastic procedures in England and further studies should be undertaken to compare the availability of these procedures in Wales, Scotland, and Northern Ireland where the healthcare is devolved.

## 5. Conclusion

This study shows that there exists a postcode lottery for bariplastic surgery in England. There is a strong need for national guidelines on the provision of body contouring procedures following MWL for the comprehensive treatment of these patients.

## Supplementary Material

Detailed questionnaire that was sent out to PCTs in England asking about funding criteria for bariplastic surgery, conformation to the AoPS guidelines and the number of procedures being funded. Information was requested on abdominoplasty, breast reduction and augmentation, facelift, and buttock, arm, and thigh lift post bariatric surgery.
