# Supplementary material for: Funding for Postbariatric Body-Contouring (Bariplastic) Surgery in England: A Postcode Lottery
Source: Plast Surg Int. 2014 Mar 20;2014:153194. doi: 10.1155/2014/153194 (PMC3980931; doi:10.1155/2014/153194)
Supplement: Supplementary file 1 — Detailed questionnaire that was sent out to PCTs in England asking about funding criteria for bariplastic surgery, conformation to the AoPS guidelines and the number of procedures being funded. Information was requested on abdominoplasty, breast reduction and augmentation, facelift, and buttock, arm, and thigh lift post bariatric surgery. [file 153194.f1.pdf]

Index 2: Questionnaire to evaluate the funding for plastic/aesthetic surgery for loose skin following weight loss as a consequence of bariatric surgery.

Contact details

Q1.1. Please provide the name of your PCT. All of the subsequent responses that you provide to this survey should relate to the PCT that you identify below.

Q1.2. Please provide your e-mail address.

Referral Process (or Guidelines)

Q2.1. Is there a guideline which is followed for referral to plastic/aesthetic surgery for loose skin following weight loss as a consequence of bariatric surgery? (Please tick)

- Yes ↑ *Please forward it to us by the email provided.*
- No ↑

Q2.2. If there is a guideline, was the 'Action on plastic surgery' (AoPS) document, produced by the NHS modernisation agency in 2005, referred to? *I have attached the document, special attention to page 13 and 14.* (Please tick)

- Yes ↑
- No ↑
- Unsure ↑

Q2.3. How does the criteria used at your PCT compare to 'Action on plastic surgery' (AoPS) document, produced by the NHS modernisation agency in 2005? (Please tick one)

- Our referral guidelines are equal to those in AoPS in all respects ↑
- Elements of our referral guidelines are equal to those at AoPS as deemed appropriate by our PCT ↑
- Our guidelines do not match those at AoPS but are specific to our PCT ↑
- Unsure ↑
- Other (please specify) ↑

Q2.5. Please list the key criteria in your guideline for determining whether a patient is suitable for referral for aesthetic/ plastic surgery for loose skin following rapid weight loss as a consequence of bariatric surgery.

Q2.6. If, post bariatric/obesity surgery, the plastic surgery is funded, then which are the procedures that are funded? (Please circle)

- Apronectomy/abdominoplasty ↑
- Breast reduction
- Breast augmentation with implants ↑
- Breast augmentation without implants ↑
- Facelift ↑
- Buttock, arm and thigh lift

Q2.4. If there is no guidelines, then do you think a guideline would be useful in reassuring the patients and the bariatric team to know what to expect after the weight loss? (Please circle)

- Yes ↑
- No ↑

Incidence *(please give figures for **2008- 09**)*

Q3.1. How many patients have been referred for Bariatric/Obesity surgery to your PCT?

Q3.2. How many patients have had funding approved for Bariatric/Obesity surgery by your PCT?

Q3.3. Who applies for the funding for plastic/aesthetic surgery, post bariatric surgery, in your PCT? (Please tick)

- GP surgeries ↑
- Department of plastic surgery in local hospital ↑
- Bariatric unit which performed the original surgery ↑

Q3.4. How many patients have applied for Plastic/Aesthetic surgery for loose skin, hanging breasts etc, which occurred as a consequence of obesity surgery? (Under exceptional circumstances included)

- Apronectomy/abdominoplasty ↑
- Breast reduction ↑
- Breast augmentation with implants ↑
- Breast augmentation without implants ↑
- Facelift ↑
- Buttock, arm and thigh lift ↑

↑

Q3.5. How many patients have had funding approved for Plastic/Aesthetic surgery for loose skin, hanging breasts etc, which occurred as a consequence of obesity surgery? (Under exceptional circumstances included)

- Apronectomy/abdominoplasty ↑
- Breast reduction ↑
- Breast augmentation with implants ↑
- Breast augmentation without implants ↑
- Facelift ↑
- Buttock, arm and thigh lift

↑ ↑

Q3.6. How many patients referred for Plastic/Aesthetic surgery for loose skin, hanging breasts etc, which occurred as a consequence of obesity surgery, has actually been operated?

- Apronectomy/abdominoplasty ↑
- Breast reduction ↑
- Breast augmentation with implants ↑
- Breast augmentation without implants ↑
- Facelift ↑
- Buttock, arm and thigh lift ↑

Incidence *(please give figures for **2009- 10** if available)*

Q4.1. How many patients have been referred for Bariatric/Obesity surgery by your PCT?

Q4.2. How many patients have had funding approved for Bariatric/Obesity surgery by your PCT?

Q4.3. Who applies for the funding for plastic/aesthetic surgery, post bariatric surgery, in your PCT? (Please circle)

- GP surgeries ↑
- Department of plastic surgery in local hospital ↑
- Bariatric unit which performed the original surgery ↑

Q4.4. How many patients have applied for Plastic/Aesthetic surgery for loose skin, hanging breasts etc, which occurred as a squeal of obesity surgery? (Under exceptional circumstances included)

- Apronectomy/abdominoplasty ↑
- Breast reduction ↑
- Breast augmentation with implants ↑
- Breast augmentation without implants ↑
- Facelift ↑
- Buttock, arm and thigh lift ↑

Q4.5. How many patients have had funding approved for Plastic/Aesthetic surgery for loose skin, hanging breasts etc, which occurred as a squeal of obesity surgery? (Under exceptional circumstances included)

- Apronectomy/abdominoplasty ↑
- Breast reduction ↑
- Breast augmentation with implants ↑
- Breast augmentation without implants ↑
- Facelift ↑
- Buttock, arm and thigh lift ↑

Q4.6. How many patients referred for Plastic/Aesthetic surgery for loose skin, hanging breasts etc, which occurred as a squeal of obesity surgery, has actually been operated?

- Apronectomy/abdominoplasty ↑
- Breast reduction ↑
- Breast augmentation with implants ↑
- Breast augmentation without implants ↑
- Facelift ↑
- Buttock, arm and thigh lift ↑

Post Operative care

Q5.1. Please outline your PCTs care pathway for patients having aesthetic/ plastic surgery for loose skin following weight loss as a consequence of bariatric surgery.

Q5.2. Is there a formal audit at your PCT to track outcomes of patients who have aesthetic/ plastic surgery for loose skin following weight loss as a consequence of bariatric surgery? (Please circle one and specify if appropriate)

- No ↑
- Yes (Please specify) ↑

Future service provision:

Q6.1. What are your PCTs plans regarding expenditure on aesthetic surgery for loose skin following weight loss as a consequence of bariatric surgery during the next five years? (Please circle one)

- Increase expenditure ↑
- Decrease expenditure ↑
- Maintain current level of expenditure ↑
- Other, please specify (e.g. not yet decided, unsure) ↑

Q6.2. Has your PCT identified review dates for referral criteria?

Q6.3. We would welcome any further comments that you wish to add regarding aesthetic surgery for post bariatric surgery.
